# Working memory, reading ability and the effects of distance and typicality on anaphor resolution in children

**DOI:** 10.1080/20445911.2015.1005095

**Published:** 2015-02-05

**Authors:** Holly S. S. L. Joseph, Georgina Bremner, Simon P. Liversedge, Kate Nation

**Affiliations:** ^a^ Department of Psychology, Social Work and Public Health, Oxford Brookes University, Gipsy Lane Campus, Headington, OxfordOX3 0BP, UK; ^b^ Department of Experimental Psychology, University of Oxford, South Parks Road, Oxford, OX1 3UD, UK; ^c^ Department of Psychology, University of Southampton, Highfield Campus, Southampton, SO17 1BJ, UK

**Keywords:** Anaphor resolution, Children, Eye movements, Reading, Reading comprehension, Working memory

## Abstract

We investigated the time course of anaphor resolution in children and whether this is modulated by individual differences in working memory and reading skill. The eye movements of 30 children (10–11 years) were monitored as they read short paragraphs in which (1) the semantic typicality of an antecedent and (2) its distance in relation to an anaphor were orthogonally manipulated. Children showed effects of distance and typicality on the anaphor itself and also on the word to the right of the anaphor, suggesting that anaphoric processing begins immediately but continues after the eyes have left the anaphor. Furthermore, children showed no evidence of resolving anaphors in the most difficult condition (distant atypical antecedent), suggesting that anaphoric processing that is demanding may not occur online in children of this age. Finally, working memory capacity and reading comprehension skill affect the magnitude and time course of typicality and distance effects during anaphoric processing.

When we read, not only do we have to recognise each individual word, but we have to integrate each word's meaning into our ongoing representation of the sentence and into the discourse model we have created that describes the state of affairs depicted in the text as a whole (Gernsbacher, 1990; Johnson-Laird, 1983; Kintsch, 1998; Van Dijk, Kintsch, & Van Dijk, 1983). A key aspect of mental model construction involves making connections between currently read text and information that was presented earlier in the passage (Garrod, O'Brien, Morris, & Rayner, 1990; Garrod & Terras, 2000; Gernsbacher, 1989; Levine, Guzmán, & Klin, 2000), as exemplified by anaphor resolution. Following its initial introduction in a passage, each time an entity (e.g. *Mary*) is mentioned, that entity can be referred to by means of an anaphoric expression (e.g. *she*). When this happens, the reader must identify the (potential) antecedent in the preceding text and then instantiate the semantic link between the antecedent and the anaphor (Garrod & Terras, 2000; van Gompel, Liversedge, & Pearson, 2004), thus resolving the anaphor and maintaining an accurate and coherent mental model of the text. As these processes are complex and resource heavy, it is reasonable to assume that they draw on skills that take time to develop. In the experiment we report here, we monitored children's eye movements as they read text containing noun phrase anaphors. This allowed us to investigate the time course of anaphor resolution as children read; we also asked whether individual differences in anaphoric processing are associated with differences in working memory and reading comprehension skill.

## Anaphor resolution in adults

There have been a number of studies examining anaphor resolution during reading in adults. An early study by Garrod and Sanford (1977) showed that participants exhibited shorter reading times on sentences containing an anaphor (e.g. *bird*) when its antecedent was typical (e.g. *robin*) rather than atypical (e.g. *goose*). They argued that this was due to greater semantic overlap between *robin* and *bird* as compared to *goose* and *bird*. They did not find a reliable effect of distance: that is, reading times on the target sentences were not reliably longer when they were separated from the sentence containing the antecedent with an intervening sentence than when the antecedent and anaphor appeared in adjacent sentences. However, because they measured reading times at the sentence level, it was not possible to know whether readers resolved the anaphor immediately (i.e. when reading the anaphor itself) or later in the sentence, and whether effects of distance would have been observed in more fine-grained analyses.

This question was investigated by Duffy and Rayner (1990; see also Liversedge & Underwood, 1998; Rayner, Kambe, & Duffy, 2000; van Gompel et al., 2004), who conducted an eye movement study in which adults read short paragraphs containing typical and atypical antecedents that appeared near (in the previous sentence) or far (followed by an intervening sentence) from the anaphor in the text. They found significantly longer first-pass reading times on anaphors that were far from, rather than near to their antecedent and also found that if the anaphoric noun was close to the antecedent, adults showed shorter first-pass fixations on the anaphor when its antecedent was typical than atypical. Although they showed no effect of typicality on the anaphor in the far condition, there was a main effect of typicality in the post-anaphor region suggesting that although readers began the process of anaphor resolution on the target word (when linking the anaphor and antecedent was relatively easy), this process continued after their eyes had moved away from the target anaphor noun region. The different patterns of effects in the anaphor and the post-anaphor regions were interpreted to reflect two different stages in anaphor resolution, described subsequently by Garrod and Terras (2000) as an initial lexically driven, context-free stage known as *bonding*, whereby a link between the anaphor and a potential antecedent is made, followed by a later process known as *resolution,* which resolves the link with respect to the overall discourse context.

## Anaphor resolution in children

Offline research has shown that children are able to resolve anaphors encountered in text when asked explicitly to do so (Ehrlich & Remond, 1997; Oakhill & Yuill, 1986; Yuill & Oakhill, 1988, 1991), but much less is known about online anaphor resolution in children, especially in relation to the effects of typicality and distance. Although there have not been any studies examining the effect of semantic typicality on anaphor resolution as children read, younger children do show a clear developmental progression from accepting only typical exemplars as members of a semantic category to gradually accepting more atypical exemplars (Bjorklund & Thompson, 1983; Bjorklund, Thompson, & Ornstein, 1983; Meints, Plunkett, & Harris, 1999). There is also some evidence pointing to an effect of distance, but findings are inconsistent. Yuill and Oakhill (1988) asked 7- to 8-year olds direct questions about anaphors they had read in narrative texts, for example, *what does “he” stand for here?* Generally, children made more errors when the anaphor was further from its antecedent, but distance effects were much weaker when the questions were more general (e.g. *Who carried his rod to the bus stop?*). There was also one inference type (lexical cohesion) in which children gave more correct answers in the distant condition than the near condition, suggesting that the distance effect is not uniform.

Distance effects were also less than straightforward in a study by Ehrlich and Remond (1997) who found that for one text, 8- to 9-year olds showed superior anaphor resolution (proportion of errors in response to an explicit question about each target anaphor in a text) when the anaphor was near its antecedent, but for a second text, the same children showed superior performance when the anaphor was far from its antecedent. It is not clear why the pattern of results differed between texts, as unfortunately, the two texts diverged in a number of ways: the second text was more difficult; it involved more protagonists (and hence potential referents); and it contained more advanced vocabulary. In sum, although there is evidence that children are affected by the distance between an anaphor and its antecedent when processing anaphoric phrases, these effects are still not well understood. It seems likely that characteristics of the text, as well as participant characteristics, will play a role in the magnitude and nature of such effects.

It is also not clear from the studies outlined earlier whether children resolve anaphors during reading, or only when prompted to do so after questioning. Two studies have examined online anaphor resolution in children, one using self-paced reading (Ehrlich, Remond, & Tardieu, 1999) and one using eye movement methodology (Murray & Kennedy, 1988). Ehrlich et al. examined anaphoric processing over eight texts in children aged 10 years. Children read each text twice, once in full, and then for a second time sentence by sentence, pressing a key to reveal the next sentence (with the exception of the target sentence, which was split into three sections). It was during this second reading of each text that data were collected. In four of the texts, the critical sentence contained either an anaphoric noun (*these shells*) or an anaphoric pronoun (*they*). In the other four texts (which contained a concurrent secondary task of detecting inconsistencies), the anaphoric noun phrase was either consistent (*these shells*) or inconsistent (*these mushrooms*) with previous text (about the seaside). Self-paced reading times on the region of text containing the anaphor were longer when it contained a pronoun rather than a noun anaphor and when it was inconsistent rather than consistent with previous text, providing evidence that children do resolve anaphors online to some degree. However, because data were collected during the second reading of the text, and because the regions of text containing the anaphor were large, we cannot know whether this occurs immediately (i.e. on the anaphor itself) or later (one or two words downstream).

Murray and Kennedy (1988) conducted an eye movement experiment in which children aged 10–11 years read sentences and questions such as, “John was buying some clothes and Mary was visiting the zoo. Was he/she/John/Mary shopping?” Children made more regressions to an antecedent when it was far from (*John*), rather than near (*Mary*), its anaphor. However, because analyses were global, that is regression rate in general was calculated, it is not known where the children regressed from or to. Furthermore, the effect of distance interacted with response type (children made more regressions to far than near antecedents when the correct response to the question was yes) and it is not clear whether the distance effect was a result of explicit questioning rather than something that would be observed during routine reading of text.

In summary, previous studies examining children's anaphor resolution have not been able to specify (1) whether children resolve anaphors immediately during reading and (2) whether effects of typicality and distance are observed during natural reading. With the metacognitive methods used in the majority of studies, it is impossible to know whether children who answered questions correctly would have resolved those anaphors had they been left to their own devices to read the text naturally. More generally, these studies do not inform our understanding of the time course of processing as children read connected text naturally and silently (Joseph, Nation, & Liversedge, 2013). By monitoring eye movements as children read texts that contain anaphoric expressions, we shed light on whether children resolve noun phrase anaphors in narrative texts spontaneously and routinely during normal reading, and the time course in which this processing takes place. It may be less immediate than in adults, as has been demonstrated in other aspects of linguistic processing (Joseph & Liversedge, 2013; Joseph et al., 2008), and therefore, typicality and distance effects may be delayed, occurring downstream of the anaphor itself.

## Individual differences

An additional focus of the current study was to investigate whether effects of semantic typicality and distance during anaphor resolution are influenced by individual differences in working memory capacity and reading comprehension skill. Consistent with the idea that maintaining a coherent situation model is a crucial element of successful comprehension, children with reading comprehension difficulties are poor at answering questions about an anaphor previously encountered during reading (e.g. Ehrlich & Remond, 1997; Oakhill & Yuill, 1986; Yuill & Oakhill, 1988, 1991). Furthermore, working memory has been strongly implicated in reading comprehension generally, and it may be the case that limitations in verbal working memory lead to poor anaphoric processing during reading. In the next few paragraphs, we discuss how reading comprehension skill and memory might constrain anaphor resolution in children.

There is a clear relationship between reading comprehension skill and anaphor resolution; indeed anaphoric resolution is a crucial part of successful reading, ensuring local and global coherence within the ongoing discourse model. Comprehension is a complex skill, drawing on a range of resources including vocabulary knowledge, lexical access, inference making and comprehension monitoring, as well as text-linking processes (Perfetti, Landi, & Oakhill, 2005). There are substantial individual differences in these subcomponents of reading comprehension in children (Nation, 2005; Perfetti et al., 2005) and complex interactions are likely to influence an individual's anaphoric processing. For example, children with good vocabulary knowledge would be expected to access words efficiently allowing more resources for comprehension processes (Perfetti, 2007), such as anaphor resolution. Furthermore, good vocabulary knowledge, and thus a richer semantic network, might engender fast allocation of an atypical antecedent to its semantic category, resulting in an earlier typicality effect. Our question, then, was whether children with stronger comprehension skills (as measured by a standardised assessment of reading comprehension) process anaphors more efficiently online than children with lower levels of reading comprehension skill. As yet, we know very little about the relationship between online and offline comprehension. Although we might assume that children with better comprehension skills process text more quickly, it may also be that they are more purposeful readers, allowing themselves more time to process text elements that are difficult (e.g. anaphors with distant or atypical antecedents). Indeed, we know this to be the case with adolescent and adult readers (Kuperman & Van Dyke, 2011; Schroeder, 2011), and that longer reading times on critical regions of text are associated with superior comprehension of that same text in children (Vorstius, Radach, Mayer, & Lonigan, 2013). We do not yet know, however, if children with generally good comprehension skills spend longer reading difficult parts of text.

Most studies that have examined the relationship between reading comprehension skill and anaphor resolution have focused on a group of children with age-appropriate reading accuracy and fluency but with specific difficulties understanding what they read. These so-called “poor comprehenders” (Cain & Oakhill, 2007; Nation, 2005) tend to show reduced effects of distance (Ehrlich & Remond, 1997; Yuill & Oakhill, 1988, 1991) in anaphor resolution. In the study described earlier, Ehrlich et al. (1999) grouped children into good and poor comprehenders and found that poor comprehenders showed smaller distance and inconsistency effects than good comprehenders in terms of a numerically smaller increase in reading times for anaphoric pronouns than noun phrases and for inconsistent than consistent noun phrases, indicating that they were less able to integrate previously encountered information into their ongoing discourse model. It may have been that the antecedent (*shells*) was activated to a lesser degree for poor comprehenders and so the bonding process whereby a link between the anaphor and a potential antecedent is made (Garrod & Terras, 2000) was weaker. In addition, poor comprehenders may have engaged less in the later resolution process, which resolves the link with respect to the overall discourse context. This would result in smaller effects of distance and inconsistency as poor comprehenders would be more willing to accept an underspecified situation model including unresolved anaphors and so they would not work as hard to resolve more difficult anaphors.

Ehrlich et al. (1999) also observed that poor comprehenders made fewer key presses overall to allow them to view previously presented text as compared to good comprehenders. This suggests that they were less concerned with maintaining a coherent discourse model as they read, making them less likely to go back to reanalyse or recheck their interpretation. Murray and Kennedy (1988) also found that less skilled comprehenders made fewer regressions overall, in line with the Ehrlich et al data. Previous research has shown that less skilled comprehenders are less likely to look back to previous text when answering comprehension questions (Garner & Reis, 1981) and are less efficient in searching for relevant regions of text (Cataldo & Oakhill, 2000). We might therefore expect children with better comprehension skills to make more regressions back to the antecedent to resolve the anaphor in our study, particularly when the antecedent is distant or atypical.

The relationship between working memory and comprehension processes has also received much discussion in the literature. As a mental representation of what is being described in the text is being constructed, it is necessary to hold relevant information (e.g. potential referents) online in memory, while also being able to dynamically update it as new information becomes available; such processes not only allow for the construction of a coherent representation of a text when reading it but also place significant demands on working memory. Since working memory is resource limited, there is a maximum amount of information that can be activated online simultaneously, and there are large individual differences in the amount of resources available for processing and storage, and these differences have been shown to constrain reading comprehension in adults (Carpenter, Miyake, & Just, 1994; Daneman & Carpenter, 1980; Daneman & Merikle, 1996; Gathercole & Baddeley, 1993) and in children (Cain, Oakhill, & Bryant, 2004; Leather & Henry, 1994; Nation, Adams, Bowyer-Crane, & Snowling, 1999; Pimperton & Nation, 2010; Seigneuric, Ehrlich, Oakhill, & Yuill, 2000; Swanson & Berninger, 1995; Yuill, Oakhill, & Parkin, 1989).

However, there has been no direct investigation of whether there is a relationship between working memory capacity and the efficiency of online anaphor resolution in children. Furthermore, existing studies have not used a measure of working memory capacity that is independent of text characteristics manipulated in the experiment. Instead, as outlined in the studies reviewed earlier, the distance between the anaphor and its antecedent (as measured by the number of words between them) has been manipulated as a proxy for working memory demands, based on the assumption that memory demands are fewer when an anaphor and its antecedent are adjacent rather than separated by a sentence in text. Although informative, these studies do not tell us anything about variation between individuals in anaphoric processing and whether it is constrained by an individual's working memory capacity. For children with increased working memory capacity, on encountering an anaphor, potential antecedents are more likely to be available in working memory than for individuals with reduced working memory capacity (Daneman & Carpenter, 1980), which should lead to faster retrieval of the antecedent (i.e. more efficient bonding).

Working memory capacity presumably also affects the later resolution process in which a reader integrates the antecedent–anaphor link into the overall discourse context, as it is necessary to maintain and process the referent and the contextual meaning simultaneously in order to assess the referent's fit. Again, children with high working memory capacity would be expected to complete this process more efficiently. We would therefore expect to observe earlier effects of distance and typicality in high- than low-span children, perhaps on the anaphor itself. We might also expect effects to be longer lasting in low-span readers, observed in the post-anaphor or antecedent regions. Alternatively, we might observe effects of typicality and distance only in high-span children, if low-span children do not have potential antecedents available to them (and therefore do not engage in the formation of a co-reference relation resulting in no additional cost to processing for atypical and distant anaphors).

In summary, it is clear that there is a paucity of research examining anaphoric processing in developing readers. There is some indirect evidence that less skilled comprehenders are poor at resolving anaphors and that this might be related to limitations in verbal working memory as well as reading skill, but more direct evidence from online methodologies is lacking. There has been no investigation of spontaneous anaphoric processing in children during natural reading; clearly however, online data are critical if we are to understand (1) how and when anaphoric devices are dealt with during reading and (2) individual differences in anaphoric processing and their relationship to reading comprehension skill and verbal working memory. To address these issues, we monitored children's eye movements as they read narratives containing anaphors, manipulating both distance and semantic typicality. This allowed us to examine the time course of anaphor resolution in children and relate this to individual differences in verbal working memory and reading comprehension. We tested typically developing 10- to 11-year olds who were fluent readers, and therefore accustomed to encountering anaphoric noun phrases during reading, and hence were able to display natural reading behaviour in our experiment; also, our group of participants were the same age at which previous studies have found online distance effects in anaphor resolution (Ehrlich et al., 1999; Murray & Kennedy, 1988).

We used a 2 × 2 repeated-measures design in which we manipulated both the distance and the typicality of an antecedent in relation to its anaphor. Specifically, we manipulated the typicality of the antecedent such that it was a typical (e.g. *truck*) or an atypical (e.g. *crane*) instance of the referring category (e.g. *vehicle*) that formed the anaphor downstream. We also manipulated the distance in the text between the anaphor and its antecedent such that the anaphor was near (in the subsequent sentence) or far (with an intervening sentence between the anaphor and antecedent) from its antecedent (see Table 1 for an example of our experimental stimuli). Our three regions of interest were the anaphor itself, the word (or words) following the anaphor and the antecedent.

**TABLE 1:** Example stimuli

	Near condition	Far condition
Typical condition	It had been a long day. The builders were exhausted. Eventually a truck arrived to help. They needed the vehicle because the load was so heavy. At last they could start work on the building.	It had been a long day. Eventually a truck arrived to help. The builders were exhausted. They needed the vehicle because the load work on the building.
Atypical condition	It had been a long day exhausted. The builders were exhausted. Eventually a crane arrived to help. They needed the vehicle because the load was so heavy. At last they could start work on the building.	It had been a long day. Eventually a crane arrived to help. They needed the vehicle because the load was so heavy. At last they could start work on the building.

The anaphor region (vehicle), post-anaphor region (because) and the antecedent region (truck/crane) are underlined.

If children respond to semantic typicality and distance manipulations as adults do (cf. Duffy & Rayner, 1990), then they would be expected to show longer reading times on the target anaphor (e.g. *vehicle* in Table 1) and in the region following the anaphor (*because* in Table 1) when the antecedent is an atypical exemplar of the semantic category represented by the anaphor and when the anaphor is separated from its antecedent by an additional sentence (i.e. when the anaphor is far from rather than near to its antecedent). We might infer that an early effect on the anaphor itself reflects an early stage of semantic association or bonding. If children attempt to fully resolve the anaphor in relation to the wider discourse context, then we might also expect more regressions to, and longer reading times on, the antecedent when it is atypical (*crane*) than typical (*truck*), and far from, rather than near to, its anaphor in the text. Effects on the post-anaphor region (*because*) could be attributed to either the bonding or resolution stage: while assumed to reflect resolution in adult readers, children have been shown to exhibit delayed disruption to processing compared to adults during reading of connected text (e.g. Joseph & Liversedge, 2013) so bonding may still be in progress at this point.

Turning to individual differences, if lower working memory capacity is associated with less activation of the antecedent on encountering the anaphor, then we would expect that children with high working memory spans would engage in faster bonding and resolution, and hence show effects of typicality and distance in early measures. Correspondingly, children with lower spans should exhibit less immediate disruption to processing, apparent in later reading time measures on the anaphor, in the post-anaphor region, or perhaps on the antecedent if they need to make an additional fixation on the no-longer-activated antecedent. They may even show no effects of typicality or distance if even typical and near antecedents are not activated. Assuming poor comprehension is associated with less drive for coherence, and in line with previous findings (Ehrlich et al., 1999), we anticipated that children with good comprehension skills would show larger effects of typicality and distance on the antecedent due to a greater need to resolve all anaphors, even difficult ones. In contrast, we predicted that children with lower comprehension skills would show fewer regressions to re-inspect the antecedent as a function of its distance or typicality (or, perhaps, show no effects at all), due to a greater willingness to continue reading without resolution. It could also be that children with poorer comprehension skills show evidence of the initial bonding stage but not of the later, more resource-demanding, resolution stage. This might be reflected in early effects of typicality and distance on the anaphor itself but no later effects on the antecedent.

## METHODS

### Participants

Fifty-one 10- to 11-year olds (Year 6) were recruited from primary schools in the Oxford area. To establish that all children had sufficient word reading skills to cope with the experiment, we screened the sample using the *Test of Word Reading Efficiency* (TOWRE; Torgesen, Wagner, & Rashotte, 1999). This requires children to read aloud as many words and non-words as possible from a list in 45 seconds. Only one child obtained a standard score below normal range and was therefore excluded from the experiment. Ten children were not tested because they were bilingual. Two children did not complete the eye movement experiment and a further eight were excluded, seven for tracker loss and one who performed poorly on the comprehension questions in the eye movement experiment (<75% correct). Data are reported for the remaining 30 children (19 girls; mean age 10.7 years, range 10.0–11.3 years).

We also administered two standardised instruments, indexing individual differences in reading comprehension and verbal working memory. In the *York Assessment of Reading for Comprehension* (YARC; Snowling et al., 2009) children are asked to read aloud two passages and then answer eight questions about each one. The questions tapped literal, inferential and vocabulary knowledge, with approximately equal proportions of the three question types in each passage. Many of the questions required anaphor resolution or the connection of ideas across the text, and therefore, performance on this test is likely to tap into similar skills required for the comprehension of our experimental items. The *Listening Span* subtest of the *Automated Working Memory Assessment* (AWMA; Alloway, 2007) is a computerised test of verbal working memory ability. Children hear a set of short unrelated sentences. They assess the validity of each sentence by responding true or false and then memorise the final word from each sentence in preparation for recalling them in correct serial order when prompted. Both tasks were administered and scored according to manual instructions. As can be seen from Table 2, mean performance was at age-appropriate levels on both measures. Children excluded showed similar scores to those included on the standardised tests.

**TABLE 2:** Mean performance of the child participants included in the experiment and those excluded on standardised tests of reading and working memory

	Standard score^1^	
Test	Children included	Children excluded
TOWRE^2^ words	102 (10)	103 (10)
TOWRE non-words	105 (15)	107 (14)
AWMA^3^	101 (14)	96 (15)^5^
YARC^4^ accuracy	106 (13)	96 (10)^5^
YARC reading rate	102 (14)	96 (18)^5^
YARC comprehension	104 (8)	100 (8)^5^

Standard deviations are in parentheses.

^1^ Standard score M = 100, *SD* = 15.

^2^ TOWRE (Torgesen et al., 1999).

^3^ AWMA (Alloway, 2007).

^4^ YARC (Snowling et al., 2009).

^5^ Note that bilingual children and the child who scored more than 1 *SD* below the mean on the TOWRE did not complete the YARC or the AWMA and therefore only 10 children's data contribute to these scores.

### Materials

Twenty-four semantic category names familiar to children were selected. Typicality ratings of exemplars were gathered from a separate group of 17 monolingual 10- to 11-year olds who did not participate in the main experiment. For each category (e.g. *flower*), we created seven possible exemplars, using adult norms (Van Overschelde, Rawson, & Dunlosky, 2004) to select two typical (e.g. *rose* and *daisy*) and two atypical (e.g. *lily*, *clover*) exemplars, which we thought children of this age would be familiar with. We also selected three distracters; one related to the category (e.g. *oak*) and two unrelated (e.g. *spaghetti* and *king*). A researcher tested children individually. Each child was presented with the semantic category printed on a large, laminated card. We then gave the child seven smaller cards, each with one of the possible exemplars printed on, along with the instruction:

I am going to give you a big card with a word on it. Then I am going to give you seven smaller cards with words on them. Your task is to decide which of the words on the smaller cards are examples of the word on the big card.

There was one practice item (*food*) with feedback. Children were able to ask questions throughout, including the meaning or pronunciation of any words they did not know but they were not told whether a word was in a semantic category.

The order of responses was recorded and coded as well as the final cards selected for each semantic category. We then selected typical exemplars which were selected first and second on 50% or more of occasions across all children, and atypical exemplars which were selected as final or penultimate (correct) responses on 50% or more of occasions. Semantic categories for which (typical or atypical) exemplars were selected on less than 50% of occasions were excluded. This left us with 16 items for which there was a clear “typical” and a clear “atypical” exemplar.

Exemplars were embedded in 16 experimental texts (see Table 1). The typical and atypical antecedents did not differ in length (*t* < 1; *p* > .6) or child frequency using ratings from written texts for children from the Oxford Children's Corpus (Wild, Kilgraff, & Tugwell, 2012; *t*(14) = 1.48, *p* = .16).

### Apparatus

Children's eye movements were recorded using a desktop Eyelink 1000 eye tracker (SR Research, Mississauga, Canada) as they read sentences from a computer monitor at a viewing distance of 62 cm. Each character covered 0.24° of horizontal visual angle and eye movements were monitored at a rate of 1000 Hz to produce a sequence of fixations with start and finish times. Although children read binocularly, only the movements of the right eye were monitored.

### Procedure

Testing took place in a quiet room close to the children's classroom. First the children completed the TOWRE in order to determine whether they met the inclusion criteria (see Participants section). This took less than five minutes. Next, the children completed the eye movement experiment. For this part, children sat in a customised chair in front of a computer monitor, supported by a chin rest and a forehead rest to ensure comfort and to minimise head movements. They first undertook a calibration procedure during which they looked at each of nine fixation points on the computer screen. They then looked at a fixation box on the left of the screen and the sentence appeared contingent on their gaze. Children were told that they would be reading a series of paragraphs displayed on the computer monitor in front of them and that they were to read each paragraph for comprehension in order to answer occasional questions. They were instructed to press a button on a handheld gamepad controller when they had finished reading each text. The button press terminated the display. If the child did not press the button within 50 seconds of the text appearing, the display was automatically terminated. Children were asked to respond to yes/no comprehension questions which followed a third of the passages by pressing one of two buttons on the gamepad. The questions were included to encourage children to read carefully; however, an accurate response did not rely on resolution of the anaphor. Paragraphs were presented in a pseudorandom order so that each child only saw one of the four versions for each stimulus set, but read an equal number of paragraphs of each version. Each child read a total of 16 experimental paragraphs, which were embedded in a larger list of stimuli containing three practice paragraphs and 10 fillers. Filler paragraphs were similar in length, writing style and content to the experimental paragraphs, but they did not contain noun anaphors. The experimental session lasted approximately 15–20 minutes. Finally, children completed the YARC (see Participants section), which took 10–20 minutes. Testing took between 30 and 45 minutes in total.

## RESULTS

Each passage was divided into three regions of interest: the anaphor (*vehicle* in Table 1), the post-anaphor region (the one or two words following the anaphor; *because* in Table 1) and the antecedent (*truck* or *crane* in Table 1). Fixations longer than 1200 ms and shorter than 80 ms were excluded from the data set. We selected a number of eye movement measures that are thought to reflect early and late stages of processing, based on previous studies (e.g. Joseph & Liversedge, 2013). The following eye movement measures were calculated for the anaphor and the post-anaphor regions: first fixation durations (the duration of the first fixation made in a region); gaze durations (the sum of all fixations in a region until a saccade out of the region); regression probability (the probability of making a leftward eye movement out of a region before leaving that region to the right); go past times (the sum of all temporally contiguous fixations including fixations after a regressive eye movement to the left of the region, until the point of fixation progresses to the region to the right) and total reading times (the sum of all fixations in a region). Regressions in (the probability of making a leftward eye movement into a region having already left that region to the right) and total reading times were also examined in the antecedent region. As is usually the case with eye movement data, our data were not normally distributed so we log transformed all the reading time data, which resulted in more normal distributions. The pattern of effects was always the same as with the untransformed data. However, for transparency, we report the untransformed means and standard deviations (*SD*s) in Table 3.

**TABLE 3:** Mean reading times and regression probabilities in the anaphor, post-anaphor and antecedent regions

Region	Measure	Typical—near	Atypical—near	Typical—far	Atypical—far
Anaphor region	First fixation durations	225 (*66*)	238 (*86*)	226 (*87*)	233 (*89*)
	Gaze durations	289 (*151*)	304 (*139*)	278 (*130*)	291 (*144*)
	Regressions out	0.23 (*0.4*)	0.15 (*0.36*)	0.31 (*0.46*)	0.27 (*0.44*)
	Go past times	380 (*225*)	367 (*215*)	409 (*281*)	427 (*322*)
	Total times	348 (*220*)	355 (*226*)	392 (*379*)	346 (*189*)
Post-anaphor region	First fixation durations	237 *(86)*	241 *(77)*	252 *(98)*	268 *(135)*
	Gaze durations	351 *(179)*	352 *(188)*	375 *(190)*	403 *(221)*
	Regressions out	0.11 *(0.32)*	0.21 *(0.41)*	0.14 *(0.35)*	0.08 *(0.27)*
	Go past times	454 *(384)*	522 *(480)*	518 *(409)*	463 *(306)*
	Total times	427 *(242)*	491 *(320)*	504 *(356)*	466 *(305)*
Antecedent region	Regressions in	0.17 (*0.38*)	0.26 (*0.44*)	0.23 (*0.42*)	0.25 (*0.43*)
	Total times	505 (*393*)	544 (*419*)	495 (421)	489 (*360*)

Standard deviations in parentheses.

Eye movement data were analysed in the R computing environment (R Development Core Team, 2012) using linear mixed models (Baayen, 2008; Jaeger, 2008; Quené & Van den Bergh, 2008). Specifically, we ran 36 models, one with each of the eye movement measures of interest in each region of interest (anaphor region, post-anaphor region and the antecedent) as the predicted variable. For each measure, we ran two models, one with the two experimentally manipulated fixed factors (typicality and distance) and the same model again but including working memory and reading comprehension skill as additional fixed effects, with random intercepts for participants and items and random by-participant and by-item slopes for all fixed effects (i.e. a full random slopes structure—see Barr, Levy, Scheepers, & Tilly, 2013).

When a model did not converge (mostly models including working memory and reading comprehension), we first took out interactions between random slopes and then removed random slopes one by one (removing those that accounted for the least variance) until the model converged. In most cases, the pattern of effects was identical between the converged model and the full unconverged model. We report only the converged models.

We centred all fixed effects using contrast coding to reduce the effects of co-linearity between the main effects and the interactions and in order that main effects were evaluated as the average effects over levels of the other predictors. This meant that there was no reference variable. Regression coefficients, standard errors (*SE*) and *t* (reading time measures) or *z* (regression probabilities) values are reported. Following Vorstius et al. (2013), we used the two-tailed criterion (*t* or *z* ≥ 1.96 *SE*), corresponding to a 5% error criterion for significance for all tests.

Summary data for each region and eye movement measure are shown in Table 3. For clarity and succinctness, we present analyses separately for each region. For each region, the simple model, including only typicality and distance as fixed effects is presented first, followed by the model including working memory and reading comprehension skill. Reading comprehension skill and working memory span were moderately correlated (*r* = .46; *p* = .011).

### Anaphor region

#### Typicality and distance effects

There were no reliable effects of typicality or distance, and no interactions between them in first fixation durations, gaze durations, go past times or total reading times (all *t*s < 1.96). There was an effect of typicality (*b* = 0.79, *SE* = .40, *z* = 1.97) and distance (*b* = 0.64, *SE* = .28, *z* = 2.26) on the frequency of regressive eye movements made out of the anaphor region to re-read previous portions of the text. This indicated more regressions out of the anaphor region when the antecedent was typical than atypical, which is in the opposite direction to that predicted and more regressions out of the anaphor when it was far from rather than near its antecedent. There was no interaction between distance and typicality (*b* = 0.33, *SE* = .57, *z* = 0.58).

#### Individual differences

**Figure 1.**
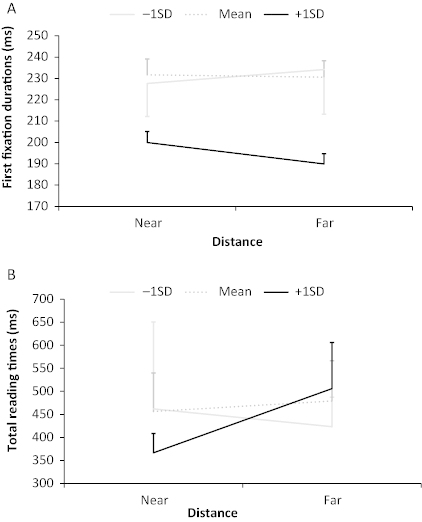
First fixation durations in the anaphor region (panel A) and total reading times in the post-anaphor region (panel B) in the near and far conditions for children with working memory scores 1 *SD* above the mean, 1 *SD* below the mean and mean scores. Error bars show *SE*.

In first fixation durations, although there were no main effects of working memory or reading comprehension skill (*t*s < 1), there was an interaction between distance and working memory (*b* = 0.03, *SE* = .01, *t* = 2.46), such that high, but not low, working memory capacity was associated with an inverse effect of distance. Figure 1A shows that high-span children (1 *SD* above the mean; *n* = 4) showed shorter first fixations overall than their peers but also longer fixations following near than far antecedents, compared to their peers. This may reflect very early bonding in the near condition for high-span readers. There were no other reliable effects or interactions in first fixation durations, gaze durations, regressions, go past times or total reading times (all *t*s and *z*s < 1.96).

In summary, analysis of the anaphor region showed effects of typicality and distance on the proportion of regressions made out of the region to re-read previous portions of the text. The effect of typicality was in the opposite direction to that predicted: children made more regressions out of the anaphor when the antecedent was typical than when it was atypical. Although unexpected, this result fits with a two-stage model of anaphor resolution, which we will consider in the Discussion section. The distance effect was in line with predictions, showing more regressions when the anaphor was far from its antecedent in the text. Furthermore, in first fixation durations, distance interacted with working memory such that children with relatively good working memory capacity made longer first fixations on the near than far anaphors, perhaps reflecting very early bonding. There was no significant relationship between reading comprehension skill and reading times.

### Post-anaphor region

#### Typicality and distance effects

There were no reliable effects of typicality or distance and no interactions between them in first fixation durations or gaze durations (*t*s < 1.96), although there was a tendency for longer gaze durations when the anaphor was far from the antecedent (*b* = 0.10, *SE* = .05, *t* = 1.94).

Although there were no main effects in the number of regressions made out of the post-anaphor region (*z*s < 1.96), there was a significant interaction between distance and typicality (*b* = 1.56, *SE* = .62, *z* = 2.49): in the near condition, children made more regressions when the antecedent was atypical than typical (*b* = 0.85, *SE* = .41, *z* = 2.02), but there was no difference in the far condition (*b* = 0.63, *SE* = .45, *z* = 1.40). Note also that there were more regressions when the anaphor was near than far from the antecedent when the antecedent was atypical (*b* = 1.19, *SE* = .45, *z* = 2.64), but not when it was typical (*b* = 0.37, *SE* = .45, *z* = 0.83). This was contrary to our prediction of increased processing difficulty for anaphors far rather than near their antecedents. There were no effects of typicality or distance in go past times or in total reading times (all *t*s < 1.96).

#### Individual differences

There were no main effects of working memory or reading comprehension and no interactions in first fixation durations (all *t*s < 1.96). There were, however, reliable effects of reading comprehension skill in all later reading time measures: gaze durations (*b* = 0.08, *SE* = .03, *t* = 2.52); go past times (*b* = 0.08, *SE* = .04, *t* = 2.27); and total reading times (*b* = 0.08, *SE* = .04, *t* = 1.99), with higher comprehension scores associated with faster reading times. There were no main effects of working memory in these reading time measures (all *t*s < 1.96). The effect of distance in gaze durations that was marginal in the simple analyses became significant (*b* = 0.11, *SE* = .05, *t* = 2.08), showing longer reading times when the anaphor was far compared to near its antecedent. There was also an interaction between working memory capacity and distance in total reading times (*b* = 0.05, *SE* = .02, *t* = 2.47), showing that high-span children showed longer reading times when the antecedent was far rather than near its anaphor compared to their peers (see Figure 1B). There were no other interactions in any reading time measure (*t*s < 1) and no effects of working memory or reading comprehension skill in the proportion of regressions made out of post-anaphor region (all *z*s < 1.96).

Overall in the post-anaphor region, children made more regressions when the antecedent was atypical than typical but only when it was close in the text to its anaphor. There was no evidence of typicality effects when the anaphor was far from its antecedent and indeed Table 3 shows that mean reading times and regression probabilities patterned in the opposite direction. There was evidence of distance effects, marginal in the simple model, but significant in the full model, with longer gaze durations when the anaphor was far from rather near the antecedent. Furthermore, children with higher working memory capacity showed larger effects of distance in total reading times, in contrast to the pattern of effects in first fixation durations in the anaphor region, but in line with predictions. Good comprehension was associated with shorter reading times, which was not the case in the anaphor region.

### Antecedent region

#### Typicality and distance effects

Children made marginally more regressions into the antecedent when it was an atypical exemplar of the semantic category (*b* = 0.62, *SE* = .33, *z* = 1.89). There was no effect of distance and no interaction (*z*s < 1.96). There were no reliable effects of typicality or distance and no interaction between them in total reading times (*t*s < 1) in this region.

#### Individual differences

**Figure 2.**
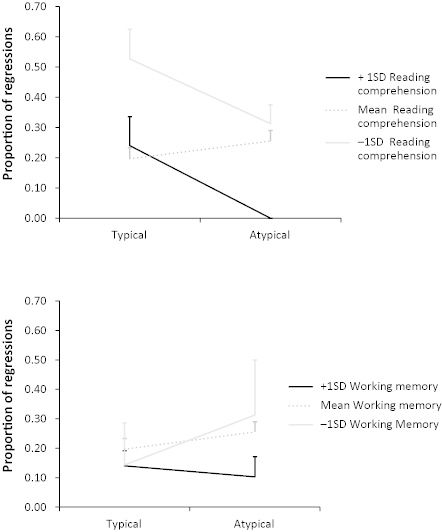
Proportion of regressions made into the typical and atypical antecedent region for children with reading comprehension scores 1 *SD* above the mean, 1 *SD* below the mean and mean scores (top panel); and for children with working memory scores 1 *SD* above the mean, 1 *SD* below the mean and mean scores (bottom panel). Error bars show *SE*.

There were no effects of working memory or reading comprehension skill on the number of regressions made into the antecedent (*z*s < 1), and a marginal effect of reading comprehension skill (with longer reading times associated with poorer reading comprehension skill*; b* = 0.08, *SE* = .04, *t* = 1.89), but not working memory (*t* < 1), on total reading times in the antecedent region. There was, however, a three-way interaction between working memory, reading comprehension skill and typicality in the proportion of regressions made (*b* = 0.13, *SE* = .06, *z* = 2.09). In this model, the effect of typicality was also significant (*b* = 0.62, *SE* = .27, *z* = 2.31). We were not able to conduct formal statistical analyses to fully explore this interaction due to our small sample size and the continuous nature of two of the variables. However, we did examine the nature of this interaction graphically by plotting reading times for those children who scored 1 *SD* above (*n* = 4) and below (*n* = 2) below the mean on the AWMA and on the YARC (above: *n* = 3; below: *n* = 2) alongside mean scores. Figure 2 shows that although children with good comprehension skills and good working memory generally made numerically fewer regressions than those with weaker skills, the pattern differed across the two typicality conditions. Those with low working memory capacity made more regressions to the atypical than typical antecedent, whereas high-span children did not show this effect. However, both children with good and poor reading comprehensions skill made more regressions to the typical than atypical antecedent. This may suggest that high-span children had already resolved the anaphor, whereas those with lower spans were still in the process of resolving it. In contrast, reading comprehension skill did not distinguish reading patterns at this stage, perhaps suggesting that making a regressive saccade to the antecedent reflects the role of working memory in identifying the antecedent (possibly reflecting bonding), but the effect of reading comprehension skills is less relevant at this point. Note, though, that due to the small numbers of children contributing to these data, these patterns must be interpreted with caution.

**Figure 3.**
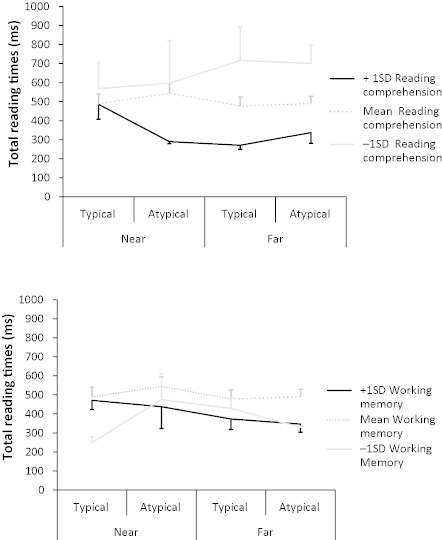
Total reading times in the antecedent region in typical and atypical and near and far conditions for children with reading comprehension scores 1 *SD* above the mean, 1 *SD* below the mean and mean scores (top panel) and for children with working memory scores 1 *SD* above the mean, 1 *SD* below the mean and mean scores (bottom panel). Error bars show *SE*.

In total reading times, there was a four-way interaction between typicality, distance, reading comprehension skill and working memory (*b* = 0.04, *SE* = .02, *t* = 2.08; see Figure 3). Again, we were unable to explore this interaction statistically, but Figure 3 suggests that those children with good reading comprehension and high working memory capacity showed little evidence of distance effects but some evidence of inverse typicality effects when the antecedent was close to its anaphor. In contrast, those children with smaller working memory capacity showed longer reading times on atypical than typical antecedents when the antecedent was near its anaphor. Children with poorer comprehension skills also showed longer reading times in the far than near condition. Although caution is needed due to the small number of children, the pattern of results in total reading times suggests that higher span readers with better comprehension skills had already resolved the anaphor at this point whereas their lower span, lower comprehending peers had not. There were no other effects (*t*s and *z*s < 1.96).

Overall, the results from the antecedent region show no reliable effects of typicality or distance, although children did make numerically more regressions into the region when the antecedent was atypical compared with typical; this was statistically significant when working memory and reading comprehension skill were included in the model. There was also a non-significant tendency towards shorter reading times for children with better comprehension skills. Interactions suggested that the combination of reading comprehension skill and working memory affected the pattern of typicality and distance effects such that those with smaller working memory capacity and poorer reading skills needed more time than their peers to complete the process of anaphor resolution, though we are reluctant to form firm conclusions in relation to these effects due to the limited size of our data set. We will consider these effects in more detail in the Discussion section.

## DISCUSSION

This experiment investigated children's online processing of anaphoric noun phrases during reading. Previous studies have relied on a secondary task or asked explicit questions to infer children's understanding of anaphor. By contrast, our data address children's spontaneous processing of anaphors during natural reading. We investigated whether children would show effects of typicality and distance, and whether these effects would be modulated by individual differences in verbal working memory capacity and reading comprehension skill.

We found effects of both typicality and distance relatively early in processing (i.e. on the anaphor itself). Children made more regressions out of the anaphor region when the antecedent was typical, contrary to predictions, and when the anaphor was far from its antecedent in text, in line with predictions. We observed an interaction between typicality and distance in the post-anaphor region such that children showed more regressions in the atypical than typical condition but only when the anaphor was near its antecedent in the text. Children also made more regressions in the near than far condition, but only when the antecedent was atypical. Although we saw no reliable effects in the antecedent region in the simple analyses, we did see more regressions to the atypical than typical antecedent when we included working memory and reading comprehension skill in the model.

The pattern of results in the anaphor region was unexpected and differed from the pattern observed for adult readers (Duffy & Rayner, 1990), showing an inverse typicality effect (Garnham, 2001). This is usually observed in adults only in later reading measures when there is no functional justification for the anaphor. For example, if an antecedent is typical and close to its antecedent, it will be easy to identify, so a noun phrase anaphor may not add new information leading to confusion (and possibly searching for an alternative antecedent) and inflated reading times (van Gompel et al., 2004). However, in children it is possible to interpret our finding as reflecting children's early anaphoric processing. On this view, at this point in processing, children use bottom-up, context-blind lexical cues (Garrod & Terras, 2000) to link the anaphor to the typical antecedent but not the atypical antecedent. This is because only the typical antecedent is activated in memory, due to its closer featural overlap with the anaphor. We had predicted more regressions and longer reading times in the atypical condition. However, the inverse effect makes sense if children (unlike adults) no longer have the atypical antecedent activated at all, then there will be no processing cost associated with reading it.

Interestingly, the pattern of typicality effects in regressions in the post-anaphor region was in the predicted direction, when the anaphor was close to its antecedent. This suggests that more children needed to make a regression, or in terminology used by Ehrlich et al. (1999), more “look backs”, before they could successfully forge a link between the atypical than typical antecedent and the anaphor. Looking across the anaphor and post-anaphor region, we can see the progression of anaphoric resolution as children read. Evidence for having resolved the easiest (least resource-heavy) antecedent–anaphor link during the initial bonding stage (Garrod & Terras, 2000) is apparent as children read the anaphor itself. When the bonding process is more difficult, as in the atypical condition, evidence of processing emerges later in reading. Although not reaching significance across all eye movement measures, there is a clear trend for increased processing times in the atypical condition when the antecedent is near, but in the typical condition when the antecedent is far from its anaphor (indeed, reading times in the atypical/far condition are as short as those in the easiest typical/near condition). Strikingly, we do not see any evidence that children are resolving the anaphor at all in the most difficult condition. It may be that the process of linking an anaphoric noun to an antecedent that is atypical and far away is simply too demanding for children of this age.

This is not to say, however, that children would not be able to resolve the anaphors in the most difficult condition offline, that is, with explicit questioning. Children may be engaging in somewhat shallow processing during reading, and so relatively difficult anaphors are not resolved as they would require too many processing resources. However, when forced to do so through questioning, a child may have gleaned sufficient information to engage in the additional processing needed to reason and answer the question correctly. This explanation would fit well with previous offline studies that have shown successful anaphor resolution (i.e. correct responses to direct questions about the anaphor) in children even when this is difficult (Ehrlich & Remond, 1997; Yuill & Oakhill, 1988). Further research that uses both online (e.g. eye movements) and offline (e.g. explicit questioning) measures of comprehension in relation to the same text would address this possibility (see Wonnacott, Joseph, & Nation, in press).

The effects of distance in the anaphor region were in the predicted direction: children made more regressions out of the anaphor when it was separated from its antecedent by an extra sentence. This result complements previous research showing that distance has a substantial effect on children's anaphor resolution (Ehrlich & Remond, 1997; Murray & Kennedy, 1988; Yuill & Oakhill, 1988). However, in the post-anaphor region, children showed no effect of distance when the antecedent was typical but an inverse distance effect when the antecedent was atypical. As described earlier, this suggests that linking a distant atypical antecedent to its anaphor may have been too difficult for children of this age and they did not begin the anaphor resolution process in this condition.

As seen in adult readers (Duffy & Rayner, 1990), it may also be the case that the time course of distance effects during anaphoric resolution is different to that of semantic typicality. The effect of distance was observed immediately on the anaphor following both typical and atypical antecedents and lingered only in gaze durations and then regressions out in the atypical condition in the post-anaphor region. We could therefore describe the effect as relatively rapid and brief. In contrast, we saw more regressions only for typical antecedents on the anaphor itself, and only in the following region did we see inflated regression rates for atypical compared to typical antecedents. We also saw some evidence of the effect of typicality continuing in regressions back to the antecedent, at least for some readers. We could, therefore, describe the effect of semantic typicality as gradual and more protracted than the effect of distance. In this way, we can clearly see a difference in the time course of distance and typicality effects in anaphor resolution in children. Nevertheless, it is clear that children of this age begin anaphoric processing on the anaphor itself, contrary to syntactic ambiguity detection (Joseph & Liversedge, 2013) and the detection of thematic implausibilities (Joseph et al., 2008) during reading, possibly because the initial bonding process is less resource heavy and requires less computation than syntactic parsing or thematic role assignment.

It is important to note that we did not observe robust effects in the antecedent region (although a non-significant trend towards more regressions made into the atypical than typical antecedent was significant in the full analyses), showing that children, unlike adults (Rayner et al., 2000), do not make reliably more regressions back to the antecedent when it is atypical or far from the anaphor. There are (at least) three possible explanations for this. First, children may have intended, but failed, to regress back to the antecedent. They may have failed because they had difficulty targeting their long-range saccades accurately, or because they were inefficient searchers (Cataldo & Oakhill, 2000), due perhaps to a poorer memory representation of where the antecedent was located within the text. However, given that we did not observe any effects in total reading times (this measure would include reading times following a mislocated regressive saccade to an adjacent region), this seems unlikely.

A second explanation is that children, or at least some of the children, did not fully resolve the anaphor online, and so the pattern of eye movements (in the anaphor and post-anaphor regions) observed reflects incomplete anaphoric processing, possibly equated to an early bonding stage. Of course, we cannot know for certain whether children fully resolved the anaphor with our current data. In order to know this, we would need to ask a question after every text for which a correct answer relied on successful anaphor resolution. Future studies might seek to do this, allowing them to investigate whether a particular pattern of eye movements (e.g. regressing back to the antecedent when it is atypical or far from the anaphor) is associated with successful anaphor resolution and whether full resolution occurs only after explicit questioning.

The final explanation for the lack of robust effects in the antecedent region is that children did not need to go back to re-read the antecedent to resolve the anaphor successfully: it was activated to a sufficient degree at the point at which they encountered the anaphor that they were able to access it, link it to the anaphor, fully resolve it and integrate this information into their discourse model. It is also possible that children made non-linguistically targeted regressions as a way of giving themselves some “time out” to engage in additional processing to resolve the anaphor (Mitchell, Shen, Green, & Hodgson, 2008), accounting for the lack of reliable effects in the antecedent region. Note that the mean regression rate into the antecedent was only 23%, so most of the time, children proceeded without a need to look back to the antecedent. Perhaps a more interesting question then is under what conditions do children need to make a regression in order to resolve an anaphor successfully? It is probably fair to say that the factors affecting whether readers do or do not make a regression when they experience processing difficulty are not currently well understood. This is true in relation to adult readers as well as child readers. Nevertheless, it is reasonable to assume that both text and reader characteristics are likely to influence regression behaviour, and it is reasonable that working memory and reading skill will play a role.

Our second main question was whether individual differences in verbal working memory and reading comprehension would affect the time course of the typicality and distance effects. We discuss each of these in turn. We did see an interaction between working memory and distance in the very first fixation made in the anaphor region: high working memory capacity was associated with inverse distance effects, that is, higher span children spent longer fixating the anaphor when it was close to its antecedent in text. This pattern of effects was reversed in total reading times in the post-anaphor region, however, where high-span children spent longer reading when the anaphor was far from its antecedent in the text. The early effect observed suggests that the antecedent remained activated in high-span children when they fixated the anaphor if it was located only a few words downstream from its antecedent in text, but this was not the case for the lower span children. It is likely that even high-span children did not have the far or atypical antecedents activated at this point. That the opposite effect is observed later (in the post-anaphor region) and in a later processing measure for higher span children only may suggest that we underestimated the time course of resolution processes even in high-span children of this age, or indeed that rather than showing delayed effects of typicality and distance, low-span children did not engage in bonding/resolution processes at all. Note that working memory capacity did not affect reading times or regression probabilities in general. This is in line with previous research with skilled readers (Kuperman & Van Dyke, 2011).

Reading comprehension skill affected reading times in the post-anaphor region with good comprehension associated with shorter reading times. It had no effect on early reading times and no reliable effect in the anaphor or antecedent region. As suggested in the introductory paragraphs, this may be because the effect of comprehension skill emerged only when comprehension was easy; children with poorer comprehension were generally slow readers (presumably reflecting generally less efficient comprehension processes), whereas children with better comprehension ability slowed down on the anaphor and antecedent (i.e. regions which required extra processing) resulting in similar reading times to their relatively poor comprehending peers. Slow reading due to generally poorer comprehension skills was therefore indistinguishable from slow reading due to the purposeful allocation of additional processing time in these regions. However, the most interesting and complex pattern of effects emerged in the antecedent region, where interactions between reading comprehension skill, working memory, typicality and distance hinted at a difference in time course of typicality and distance effects for those with good and poor reading comprehension skills and high and low working memory capacity.

Although our data set was too small to conduct extensive analyses examining the different patterns of effects for different profiles of readers, we can speculate that those with both high working memory capacity and good comprehension skills resolved the anaphor by the time their eyes left the post-anaphor region (much like adult readers). This may have been achieved through a combination of fast bonding, aided by high working memory capacity, rich vocabulary knowledge and strong semantic/conceptual links within their lexicons; and efficient resolution, helped by a strong drive for coherence, a detailed and accurate discourse model and meticulous comprehension monitoring. Those with poorer comprehension skills and smaller working memory capacity may have still been in the process of trying to resolve the anaphor (as indexed by more regressions to the atypical antecedents for lower span children, and longer total reading times for poorer comprehenders on the atypical and distant antecedent). For these children, poor working memory or comprehension skill may have led to slower bonding, requiring a re-inspection of the atypical antecedent or more difficulty resolving the anaphor, necessitating longer reading times on the antecedent if the discourse model was underspecified or not active in memory. Our data are consistent with the possibility that working memory capacity is crucial for the early bonding stage (reflected in the interaction between working memory and distance on the anaphor itself as well as the effect in regressions back to the antecedent), whereas reading comprehension is more important for the later resolution stage, where the pattern of total reading times on the antecedent differed substantially for those with good and poorer comprehension skills. Clearly, however, further research is needed to substantiate this speculation.

Finally, it is possible that those with poor memory and comprehension skills may not have begun, or may have failed, to resolve the anaphor. This could have been due to a combination of potential referents being unavailable in memory, an impoverished discourse model and a lack of drive to improve the coherence of the reader's ongoing representation of the passage meaning. Further investigation of these issues with a much larger sample is crucial to examine this possibility further, together with the examination of online and offline comprehension processes within the same text.

Both Murray and Kennedy (1988) and Ehrlich et al. (1999) found that in general, poor comprehenders made fewer attempts to go back and re-read previous portions of the text. In our study, we examined regression probability in more detail to establish whether comprehension skill affected whether children made more regressions out of the anaphor or the post-anaphor region, and more regressions into the antecedent region. Somewhat surprisingly, we found no evidence that this was the case. Likewise, there was no relationship between verbal working memory capacity and the proportion of regressions made into or out of a region (although this is in line with previous research: Kuperman & Van Dyke, 2011). The most likely explanation for this is that regressions are not confined to our target regions and an increase in “look backs” observed in previous studies is a global effect that reflects increased comprehension monitoring and integration processes that cannot be pinned down to a specific area of text. Note also that regressions were probably made primarily out of a question in Murray and Kennedy's study (we cannot know for certain as they reported global regression rates), and look backs were button presses made in order to view a previous screen in the Ehrlich et al. study; both paradigms were therefore quite different from our study which looked at regressions during normal text reading. Nevertheless, our findings suggest that even 10- to 11-year olds with good comprehension skills do not read in the same way as skilled adult readers who do launch and target their regressive saccades from and to particular regions of text when resolving anaphors (Rayner et al., 2000) and more generally (Frazier & Rayner, 1982; Meseguer, Carreiras, & Clifton, 2002) although this point is not uncontroversial (Mitchell et al., 2008; von der Malsburg & Vasishth, 2013).

In relation to the issue of regressions, there are two more general points to note. First, children may have made a significant number of regressions due to factors other than resolving the reference relation, for example, due to reduced proficiency in basic oculomotor control during reading. As mentioned, the factors that drive regressions are not well understood, and further research specifically exploring factors that cause regressive eye movements and re-reading (both in adults and children) is required before we are able to interpret this aspect of our results in further detail. Second, it is worth emphasising that even when we do see an effect in regressions, the differences are usually based on mean regression rates of between 20% and 40% of trials. This means that on the majority of trials, participants did not have to regress to resolve the difficulty they encountered (assuming they did resolve the difficulty). Hence, the predominant behaviour is resolution without regression, and arguably this should be the situation we primarily explain, in addition to examining the circumstances under which readers do make regressions.

In conclusion, our data reveal both early and late effects of anaphor resolution during text reading in children aged 10–11 years. More regressions made out of the anaphor itself may represent an early “bonding” stage of anaphoric processing, whereas effects in the post-anaphor region and the interactions in the antecedent region may reflect full resolution of the anaphor. Strikingly, children showed no evidence of resolving the most difficult anaphors online, that is, when the anaphor followed an antecedent that was both an atypical exemplar of the semantic category depicted by the anaphor and when the antecedent was relatively distant from the anaphor in the text. Finally, our data suggest that verbal working memory and reading comprehension skills affect the time course with which the distance and the typicality of an antecedent in relation to an anaphor influence anaphoric processing in children.
